# Acute HIV-1 Infection: Paradigm and Singularity

**DOI:** 10.3390/v17030366

**Published:** 2025-03-03

**Authors:** Antoine Chéret

**Affiliations:** 1 Inserm U1016, CNRS UMR 8104, Institut Cochin, Université Paris Descartes, 75014 Paris, France; antoine.cheret@aphp.fr; 2 Service Plateforme de Diagnostic et Thérapeutique Pluridisciplinaire, Centre Hospitalier Universitaire, 97159 Pointe à Pitre, Guadeloupe, France; 3 INSERM-CIC-1424, Centre Hospitalier Universitaire, 97159 Pointe à Pitre, Guadeloupe, France

**Keywords:** acute HIV infection, HIV-1 DNA, reservoir, ART viral transmission

## Abstract

Acute HIV-1 infection (AHI) is a transient period where the virus causes evident damage to the immune system, including an extensive apoptosis of CD4+ T cells associated with a high level of activation and a major cytokine storm to fight the invading virus. HIV infection establishes persistence by integrating the viral genome into host cell DNA in both replicating and non-replicating forms, effectively hiding from immune surveillance within infected lymphocytes as cellular reservoirs. The measurement of total HIV-1 DNA in peripheral blood mononuclear cells (PBMCs) is a reliable reflection of this reservoir. Initiating treatments during AHI with nucleoside reverse transcriptase inhibitors (NRTIs) and/or integrase strand transfer inhibitors (INSTIs) is essential to alter the dynamics of the global reservoir expansion, and to reduce the establishment of long-lived cellular and tissue reservoirs, while preserving and enhancing specific and non-specific immune responses. Furthermore, some of the patients treated at the AHI stage may become post-treatment controllers and should be informative regarding the mechanism of viral control, so patients treated during AHI are undoubtedly the best candidates to test innovative remission strategies toward a functional cure that could play a pivotal role in long-term HIV control. AHI is characterized by high levels of viral replication, with a significant increase in the risk of HIV transmission. Detecting AHI and initiating early treatment following diagnosis provides a window of opportunity to control the epidemic, particularly in high-risk populations.

## 1. Introduction

Acute HIV-1 infection (AHI) is a phase marked by a high risk of transmission and intense multiplication of the virus within the host during the 12 weeks following infection. In 2023, the United Nations estimated that there were 39.9 million people living with HIV (PLWH) and around 1.3 million who had been newly infected [1]. AHI has long been recognized as a period of high transmission risk due to the elevated viral loads observed during this phase and the lack of awareness of HIV status, with the risk estimated to be up to 26 times higher than during the chronic phase [2,3,4,5]. The epidemic poses a major risk for men who have sex with men (MSM) in both developed and developing countries [1]. In 2016, the CDC estimated that approximately 4% of the new HIV infections in the United States involved transmission from a person with acute HIV [3]. AHI can present without symptoms or with non-specific, variable, and self-limiting signs, often leading to misdiagnosis as another viral infection [6]. Consequently, a high level of clinical suspicion and thorough assessment of risk factors are essential for its identification. Since 2015, the World Health Organization (WHO) guidelines have recommended initiating combination antiretroviral therapy (ART) in PLWH, regardless of CD4+ T-cell count [7]. The use of second-generation INSTIs has been increasingly recommended by experts and incorporated into current guidelines for the management of AHI [8,9,10]. Such treatments should include two nucleoside inhibitors (tenofovir disoproxil or tenofovir alafenamide and lamivudine or emtricitabine) in combination with dolutegravir [11] or bictegravir or alternatively, a boosted protease inhibitor. The combination therapy is also adjusted according to the results of genotypic antiretroviral resistance tests, tolerance, and comorbidities, with the aim of maintaining long-term virological success. Early treatment initiation during AHI promotes individual and public health benefits, including preventing AIDS (Acquired Immunodeficiency Syndrome) progression and reducing secondary HIV transmission [12]. This early intervention also positions these patients as optimal candidates for future HIV treatment strategies, as their immune system is less damaged, and the viral replication and evolution are limited. Therefore, there is a strong rationale for establishing policies to promote early HIV diagnosis, especially for patients with symptoms consistent with AHI or recent exposure to HIV, to ensure timely entry into care and prompt treatment.

This article reviews the key aspects of AHI and its treatment implications, focusing on strategies aimed at ending the HIV epidemic through remission and the pursuit of a cure, which remain significant challenges.

## 2. HIV-1 Infection and Viral Reservoir Establishment

Following mucosal HIV infection, the virus spreads via myeloid cells such as dendritic cells (DCs) and macrophages. Soon after HIV-1 exposure, these cells migrate to draining lymph nodes and transfer it to CD4+ T cells through virological synapses in the absence of a productive infection [13,14]. They allow a significant spread of viral infection to tissues, and HIV can persist for years in myeloid cells [15], highlighting their specific role in long-term viral persistence [16]. In SIV models, infected DCs reach lymph nodes within 24 h, marking the ‘eclipse stage’, a period lasting 7–21 days during which the virus remains undetectable [17,18]. During the acute phase, the virus predominantly targets CCR5+ cells, including CD4+ T cells and myeloid cells [19,20]. Although R5- and X4-tropic variants may coexist in the source, X4 variants contribute minimally to early infection [21]. However, X4 or mixed X4/dual-tropic viruses are linked to faster disease progression due to their impact on lymphocyte homeostasis [22,23]. X4 or mixed R5/X4 variants typically emerge in advanced stages, especially in patients with CD4+ T-cell counts below 200 cells/µL [24].

Phylogenetic studies of the circulating viruses in acute HIV infection (AHI) cohorts have shown that 30–50% of transmissions occur within shared clusters [25,26,27]. However, some studies indicate multiple viral founders in cases involving more permissive transmission routes, such as men who have sex with men (MSM) and intravenous drug users (IVUs) [20,28]. Interestingly, another study found a similar frequency of single-virus transmission across MSM, heterosexuals, and IVUs [29], with variations likely due to the AHI stage and immune pressure over time [12]. Typically, transmission involves a single or limited number of replication-competent virions that lead to productive infection [20,30,31]. The virus rapidly spreads to secondary lymphoid organs, including the lymph nodes, spleen, and gut-associated lymphoid tissues, which contain 98% of all CD4+ T cells and are sites of high levels of replication. This leads to multi-organ invasion, including the genital tract [32] and central nervous system [33]. The peak of HIV-RNA levels occurs within two to four weeks post-infection, establishing a ‘viral set point’ with prognostic significance approximately 31 days (interquartile range 18–42 days) after initial plasma viremia detection [34,35,36,37]. Notably, viral replication capacity in AHI influenced by the gag gene correlates with the viral set point and significantly impacts disease progression [38].

A latent HIV cellular reservoir is rapidly established after transmission through proviral integration into host cell DNA [39,40]. In AHI, virus production primarily occurs in activated CD4+ T cells, forming an ‘active reservoir’ that amplifies the amount of virus and leads to a stable, quiescent reservoir of transcriptionally silent cells that maintain long-term viral persistence [41,42]. Total HIV-1 DNA in PBMCs (HIV-DNA) is a relevant clinical marker of the reservoir size, affecting disease progression despite its inability to differentiate between replication-competent from defective virus [43]. Key reservoirs include the gut, rich in CCR5+CD4+ and TH17 cells [44,45]; the central nervous system (CNS), involving infected microglia and astrocytes [46]; and the seminal compartment, which accumulates HIV progressively during AHI [47,48]. Early AHI stages show lower HIV levels in blood and tissues [40], but reservoir levels in PBMCs during AHI are higher compared to the chronic phase, predicting disease progression [43,49]. Patients with high levels of integrated HIV-DNA shortly after infection are at a higher risk of rapid progression towards AIDS, indicating that the rapidity of progression is well determined soon after infection [50]. Integrated HIV-DNA likely accounts for most of the predictive value of total HIV-DNA observed in previous studies, as this stable form of HIV persistence plays a key role in driving the course of infection [50].

The quiescent CD4+ T-memory cells are the form of HIV reservoir that has been most studied. With an increasing expression of CCR5, we distinguish naive (TNs), central memory (TCMs), transitional memory (TTMs), effector memory (TEMs), and effector T cells [51,52]. These various lymphocyte subpopulations exhibit different half-lives, as well as distinct activation and proliferation capacity. Quiescent CD4+ T cells exhibit high infection levels in AHI [27,42,53]. Long-lived TCMs and transitional memory cells, although low in quantity, are critical to reservoir maintenance due to their long half-lives, and TCMs ensure the long-term memory of the immune response in humans [54,55]. In AHI, the TTM reservoir is dominant [27], whereas in chronic infection, TCM cells with extended half-lives play a major role in maintaining viral persistence, sustaining residual replication, and contributing to progressive CD4+ T-cell depletion [56,57].

## 3. Immune Response

During acute HIV infection (AHI), myeloid cells such as macrophages and dendritic cells represent the first line of defense [16]. Myeloid cells detect pathogens through pattern recognition receptors (PRRs) located on their membranes or within the cytosol, recognizing viral pathogen-associated molecular patterns (PAMPs). This recognition triggers the secretion of interferon (IFN)-α/β, the primary antiviral molecule, initiating the immune response against the virus [58,59]. These cells initiate the adaptive immune response in lymphoid tissues by presenting antigens and releasing cytokines and chemokines, which guide the polarization and migration of naïve T and B cells [60]. Both cell-mediated and soluble factors are generated, leading to intense immune activation with elevated plasma levels of several cytokines and chemokines, including rapid and transient increases in type I and III interferons that predict disease progression [61] and interleukin-15; a significant rise in inducible protein 10 levels; more sustained increases in tumor necrosis factor alpha and monocyte chemoattractant protein-1; slower increases in additional pro-inflammatory factors such as interleukin-6, interleukin-8, interleukin-18, and interferon-gamma; and a late rise in the immunoregulatory cytokine IL-10 levels [62]. Cytokine and chemokine levels in plasma rise with viral replication [62], which recruits and activates immune cells, including macrophages, natural killer (NK) cells, and dendritic cells. Among them, interleukin-12, interleukin-15, and interleukin-2 are powerful activators of NK cells [16]. Innate immune cells play a crucial role in the early stages of HIV infection to control viral transmission and dissemination [63,64] and modulate the adaptive immune response [16,65]. While NK cell and DC functions are impaired during chronic HIV-1 infection, they may instead be enhanced during AHI, leading to increased immune activation and viral replication [65].

Cytokine storms induce apoptosis in HIV-specific and non-specific B and T cells [65,66,67]. This response leads to the exhaustion and senescence of T cells, as well as the depletion of T and B cells [68] in the blood and gastrointestinal lymphoid tissue (GALT) [69], resulting in follicular hyperplasia, immune ‘burn out’ in lymphoid tissues [70], lymph node fibrosis, regulatory T-cell (Treg) proliferation [71], and microbial translocation, especially during chronic infection [72,73,74]. This exaggerated cytokine response likely contributes to the early immunopathology of the infection and associated long-term consequences [62]. Additionally, host restriction factors (HRFs) play a vital role in the early antiviral response. These cellular proteins suppress viral replication and dissemination by interfering with key stages of the viral life cycle, including entry, genome transcription and replication, protein translation, viral particle assembly, and release [75].

CD4+ and CD8+ T-cell responses play key roles in controlling viral replication during AHI [76,77]. CD8+ T cells initially control viremia by recognizing early-stage viral epitopes (envelope glycoprotein (Env), negative regulatory factor (Nef), group-specific antigen (Gag), protein 24 (p24), proteins coded by the Polymerase gene (Pol)) [78]; the strength and timing of the cytotoxic response influence the viral set point [37]. This response is highly specific to autologous epitopes with limited cross-reactivity and involves cytolytic activity through perforin, a key mechanism for inhibiting viral replication, as observed in HIV controllers who present strong CD8+ T-cell activity and were able to suppress HIV infection [79]. Additionally, these cells secrete β-chemokines (Regulated upon Activation, Normal T-cell-Expressed and -Secreted (RANTES), Macrophage Inflammatory Protein-1 alpha (MIP-1α), Macrophage Inflammatory Protein-1 beta (MIP-1β)), which further contribute to viral control [80]. Responses to conserved epitopes are linked to long-term control, as ‘protective’ Human Leukocyte Antigen (HLA)-I alleles (e.g., *HLA-B27, HLA-B*5701) correlate with lower viral loads and slower disease progression [81] due to the recognition of less variable viral regions (e.g., Gag) [82,83]. However, this response diminishes over time due to mutant viral escape, chronic activation, and reduced TH1 response [82]. CD8+ T cells also lose the ability to upregulate perforin after peak viremia [84,85]. Innate immune cells, including dendritic cells, monocytes, and NK cells, influence disease progression rates, and certain receptor–HLA combinations are linked with better NK function and viremic control [86,87].

The CD4+ T-cell response, particularly through its TH1 helper function, is crucial in controlling HIV infection by recruiting immune cells, aiding lymphocyte expansion, and enabling direct cytotoxicity [88]. This response, however, is significantly compromised due to early CD4+ T-cell depletion post-Fiebig I [89,90]. The beneficial impact of CD4+ T-cell function on lowering the viral set point and attenuating disease progression during AHI has been established [91,92,93]. Although both CD4+ and CD8+ T-cell responses are weak in AHI, a small percentage of HIV controllers (0.5%) maintain strong CD4 and CD8 responses, contributing to natural viral control [79,94,95]. High cytokine levels during AHI cause uncontrolled CD4+ T-cell proliferation, depleting memory cells and impeding immune compensation [96]. CD4+ T-cell count falls quickly in most patients, often dropping below normal levels (<500/mm^3^) [97]. CD4+ T-cell function declines due to rapid anergy, which results in the overexpression of the negative regulatory factor PD-1 [98], blocking of which promotes better control of viral replication [99]. The significant loss of CD4+ T helpers and continuous activation are responsible for the defective functionality of CD8+ T cells [100].

The depletion of T lymphocytes and the disruption of T-cell homeostasis presumably drive and precede the progressive loss of B cells [101], notably due to the loss of T-cell types important for the development of germinal centers, with up to 50% of these centers being lost within the first 80 days of infection. Moreover, persistent B-cell activation at the time of AHI creates large populations requiring T-cell help, which is unavailable due to the loss of antigen-specific T cells. Without this support, B cells undergo apoptosis within days [102]. High cytokine production also drives excessive B-cell activation, contributing to a reduction in B cells [101], with functional disruption leading to hypergammaglobulinemia, polyclonal anti-HIV activation [103], weaker responses to non-HIV antigens, inadequate antibody production, and diminished long-term B-cell memory [104,105]. Initial gp41 antibodies appear around day 13, gp120 antibodies on day 14, and specific IgA in mucosal tissues three weeks post-infection, although these are non-neutralizing. Neutralizing antibodies emerge at 12 weeks but are limited in targeting the transmitted virus, failing to impact evolving variants [106].

**Figure 1:**
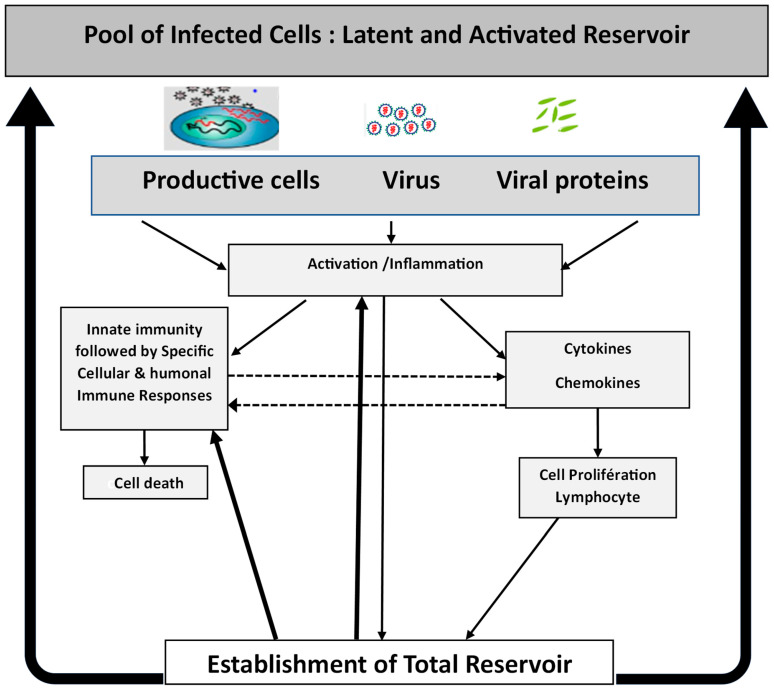
**Patho-physiological model of acute and recent HIV infection.** Acute HIV infection marks the onset of a virological and immune storm at its peak. The initial viral replication in infected productive cells leads to a high production of virions and viral proteins, which triggers first intense innate immunity response (including myeloid cells) and contribute to the immune storm by inducing activation and inflammation, with the production of inflammatory cytokines and chemokines, and mobilizing cellular and immune responses. Acute infection elicits intense immune responses, characterized by massive T cell activation and proliferation, which facilitate cell infection and the establishment of a comprehensive HIV reservoir. This reservoir includes both latently infected and productive cells within lymph nodes and tissues. A more or less attenuated level of inflammation and various immune stimulations then persist, maintaining a continuous model with constant viral replication in the absence of treatment.

These immune responses ultimately yield partial viral control, establishing a virologic set point in untreated individuals [19]. Interdependence between immune activation and the viral reservoir creates a self-sustaining cycle that is central to HIV persistence and poses a significant challenge to eradication efforts [107] (Figure 1).

## 4. Diagnosing Acute HIV Infection: A Critical Issue

Clinical symptoms, resembling a flu-like syndrome, are present in 23–92% [6,108] of patients with AHI; the proportion of asymptomatic or mildly symptomatic acute HIV infections (AHIs) remains difficult to determine, as individuals without symptoms often do not seek medical care. Most of the studies recruited symptomatic study populations and thus give a biased view of acute infection. AHI is symptomatic in over 50% of cases [109]; however, more than 90% of AHI cases remain undiagnosed [110] primarily due to the highly variable clinical manifestations [111]. Most symptoms are non-specific, and atypical clinical presentations are common. The lack of specific symptoms or a combination of symptoms with adequate diagnostic sensitivity and specificity makes the prompt diagnosis of acute HIV infection particularly challenging [6]. Symptom severity, quantity, and, in some studies, duration of AHI are associated with the rate of subsequent disease progression, especially when neurological symptoms are present [112]. The symptoms are the consequences of high levels of cellular activation and inflammation [108,113]. They usually appear within 10 to 15 days, and persist up to 10 weeks after acute infection [108,114]. HIV infection is a multi-organ disease; the main symptoms may include fever, headache, myalgia, pharyngitis, lymphadenopathy, skin rash, gastrointestinal problems, and neurocognitive disorders [114]. AHI presents a mononucleosis-like syndrome that is often accompanied by thrombocytopenia and hepatic cytolysis [11,114]. However, clinical and biological features are subject to individual variations: the intensity of symptoms is correlated with HIV-RNA [115] and HIV-DNA levels in PBMCs [116] and inversely correlated with the number of CD4+ T-cell count [113,116]. The severity of symptoms is also associated with the risk of disease progression [117,118]. Any acute viral syndrome in a patient at risk of HIV exposure should prompt consideration of an HIV diagnosis. Therefore, increased awareness among primary care physicians should be encouraged, particularly when encountering individuals from high-risk populations.

**Figure 2:**
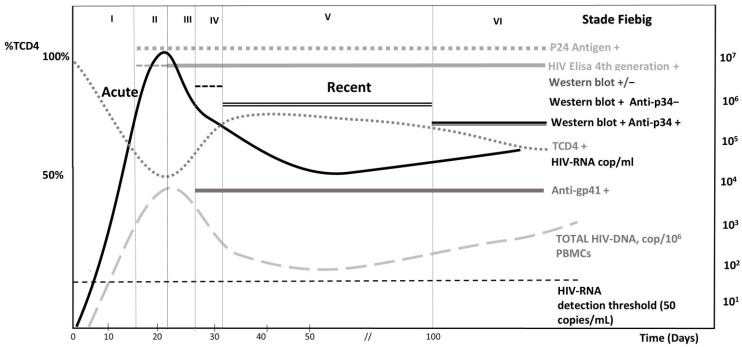
**Development of Biological Markers at the time of Acute and recent HIV Infection:** The progression of HIV-1 infection can be divided into six distinct stages named Fiebig stages based on results of standard laboratory tests. They are characterized by the sequential detection starting with HIV-1 RNA, p24 antigen and specific antibodies against HIV-1 proteins, detected by Elisa (the 4th generation, the more sensitive), including anti-p34, detected by Western blot analysis. In the very first step of acute HIV infection, the pattern of the Western blot may be indeterminate, then weak or with an isolated anti-p24 band, followed by reactivity against gp41 and progressing to a complete pattern. HIV viral reservoir can be estimated using the quantification of total HIV-DNA in PBMC, which is detectable in the first step. For newborns, the diagnosis of acute HIV infection can be established by quantifying plasma HIV-RNA in the first days of life, as well as by measuring total blood HIV-DNA. A plus sign indicates a positive test result, a minus sign denotes a negative result, and a plus-minus sign represents a borderline-positive result. Adapted from Cohen et al, NEJM, 2011 [19].

Following viral transmission, an eclipse phase of approximately ten days occurs before HIV-RNA can be detected in blood plasma. The kinetics of antibody appearance (assessed by ELISA), combined with the quantification of HIV-RNA and/or HIV-DNA levels (in newborns) [119] and the p24 antigen, have enabled the establishment of diagnostic criteria for AHI. The most widely used classification system, known as ‘Fiebig stages’ [120], is based on the kinetics of these markers. Nevertheless, a new classification may emerge with the advent of more sensitive fourth-generation ELISA tests that incorporate P24 antigen detection [121]. The number of HIV antibodies detected by Western blot (WB) correlates with the time elapsed since infection: ≤1 antibody after a median of 28 days post-infection (range: 18–49 days), 2–4 antibodies after 39 days (range: 16–118 days), ≥5 antibodies after 63 days (range: 18–241 days) [116]. The distinction between acute and early HIV infection has varied definitions, but it is commonly associated with the full development of antibody responses and the establishment of a stable viral load set point, which typically occurs 4 to 5 weeks after the onset of viremia [35]. Thus, during the very early stages of AHI, the diagnosis can be established by the presence of a negative or weakly positive HIV-1 ELISA (third or fourth generation), a negative or incomplete (≤1 antibody) Western blot for HIV-1, and a positive HIV-RNA load and/or positive p24 antigen (Fiebig stages I, II, and III). In later stages, the diagnosis of AHI may be established by a positive ELISA assay with an incomplete HIV-1 Western blot (≥2 and <5 antibodies, specifically the presence of anti-p24 antibodies along with anti-gp160, anti-gp120, or anti-gp41 antibodies) and a positive HIV-RNA load (Fiebig stages IV and V) [122]. However, immunoblots may not be effective in confirming the recency of HIV-1 infections [123]. Alternatively, rapid diagnostic tests (RDTs), which do not require laboratory infrastructure, are gaining public health interest. These tests promote access to HIV screening, providing results within 30 min, and enabling use in non-clinical ‘outside the walls’ settings for populations at risk of HIV acquisition. There are various RDTs (using immunochromatographic agglutinations) that can be achieved on different biological fluids (plasma, whole blood, or saliva). While they are less sensitive than ELISA tests, particularly when samples are taken during the seroconversion phase, and their performance is weaker with saliva samples [124], RDTs remain valuable for mass screening in high-risk populations [123]. For the earliest stages of HIV, the use of nucleic acid testing (NAT) allows viral RNA detection even before antibodies or p24 antigens are present, thereby identifying acute infections more reliably. Although studies have reported that fourth-generation ELISA tests for HIV, which combine the p24 antigen and antibody detection, are effective in identifying infection starting at Fiebig stage II, stage I infections remain omitted [125]. In clinical settings, combining fourth-generation assays with NAT could improve early detection in high-risk patients, particularly those presenting with symptoms consistent with acute HIV infection (Figure 2). This approach can help bridge the detection gap at Fiebig stage I, enabling timely diagnosis and reducing potential transmission risks [125]. According to Powers et al., more targeted and frequent testing in high-risk populations is essential for identifying acute HIV infections [126]. The need for targeted screening within high-risk groups undoubtedly requires better integration into public health responses. The revelation of serological status allows and frequently leads to a modification of sexual behavior which in turn helps reduce the risk of transmission [127]. Without these strategies, efforts to steer the epidemic toward elimination could be undermined. Early detection during the acute phase is crucial for initiating treatment to control the spread of HIV and prevent the establishment of large HIV reservoirs, which are central to long-term persistence, transmission risk, and disease progression [126]. Early and effective screening in symptomatic patients or those with recent high-risk exposure should facilitate prompt treatment, yielding proven benefits both individually and collectively.

## 5. Key Virological Considerations in Acute HIV Treatment

Early treatment during acute HIV infection (AHI) can reduce plasma HIV-RNA to undetectable levels (<50 copies/mL) within months [11,128,129,130]. Additionally, more pronounced benefits are observed when treatment is initiated earlier in AHI, including a lower viral set point and better preservation of CD4+ T-cell counts [129,131]. Despite the benefits of initiating ART during AHI, low-level viremia persists, originating from at least two cellular reservoir compartments: one where viral production gradually declines over time and another where it remains stable for at least seven years [132]. The clearance of HIV-1 infected cells is linked to the extent of viral replication, as measured by cell-associated RNA levels in both blood and lymph nodes [132]. The gut-associated lymphoid tissue (GALT), which harbors 60% of the body’s lymphocytes, plays a major role in HIV persistence and is therefore a critical target for strategies aimed at reducing this viral reservoir. [43]. HIV DNA levels in GALT decrease following the initiation of cART in AHI [133] but persist nonetheless, with variations observed across different gut sites [45,134,135]. Studies have shown that starting ART in AHI significantly impacts the HIV blood reservoir, with faster and more profound reductions compared to treatment during the chronic phase [43]. Total HIV-DNA decreases particularly within the first eight months of AHI treatment [136] and interestingly, correlates well with integrated HIV-DNA levels [137]. The OPTIPRIM-ANRS 147 and 148 clinical trials demonstrated that two years of ART significantly reduced HIV-DNA in PBMCs and rectal tissue, particularly levels in short-lived memory T cells (TTMs, TEMs). These cells, which constitute the primary reservoir, have limited proliferative capacity and shorter lifespans due to their higher susceptibility to activation and viral production [30]. Early treatment during AHI stabilizes the reservoir, preserving long-lived memory T cells (TNs, TCMs) and limiting the reservoir to short-lived cells [27,30,138,139]. This effect likely results from suppressed viral replication and reduced memory T-cell activation. Over time, ART allows HIV-DNA decline, likely reflecting the death of short-lived infected cells [136].

Conversely, in patients treated during the chronic phase, total HIV-DNA levels showed the most significant decline during the first year of ART, primarily due to the clearance of infected activated cells. However, this decline slowed thereafter, as integrated HIV-DNA in latent cells persists at high levels [140]. Early ART administration reduces the initial, labile, unintegrated forms of HIV and limits integrated forms, largely eliminating the majority of short-lived cells infected during AHI. In contrast, chronic-phase reservoirs consist primarily of stable proviruses in long-lived cells unaffected by ART [50,141]. Thus, early ART during AHI prevents the establishment of stable, hard-to-eliminate reservoirs. Studying integrated and total cell-associated HIV-DNA could help identify optimal candidates for curative strategies, potentially supporting a functional cure when combined with other therapeutic interventions. In various cohort studies and randomized trials on AHI treatment, partial or even long-term viral control was observed in some patients who discontinued treatment [142,143,144,145]. Early treatment initiation delays the onset of virologic rebound, with some studies suggesting an optimal start time of around three weeks post-infection [146,147]. Additionally, the total cell-associated HIV-DNA level has been shown to predict the timing of viral rebound following treatment discontinuation [148]. In a very limited number of individuals designated post-treatment HIV controllers (PTCs), viremia remains suppressed for prolonged periods after ART withdrawal [149]. The protection of central memory and naive CD4+ T cells is a key factor in post-treatment controllers (PTCs) who were treated during AHI [30]. The estimated probability of maintaining viral control 24 months after early treatment interruption is approximately 15% among patients who received treatment during AHI versus less than 1% of those not treated [145]. This immune protection resembles that observed in individuals with protective HLA alleles, such as HLA-B5701, HLA-B5703, and HLA-B*27, which are commonly associated with elite controllers but less frequent in human PTCs [150,151]. Similar patterns have also been noted in animal models, such as sooty mangabeys and early-treated monkeys [152]. A low HIV reservoir level, especially when predominantly found in naive and central memory CD4+ T cells, is strongly associated with favorable outcomes in patients treated during AHI [30].

Historically, HIV-1 clade B strains predominated in Europe and North America, shaping the design of diagnostic tests and treatment guidelines. However, globalization and migrations have increased viral genetic diversity in these regions, with expansion of HIV-1 non-B clades (A, C, D, and recombinants) [153]. This diversification directly impacts acute HIV infection because some non-B clades may lead to faster progression to AIDS and exhibit specific resistance patterns to some antiretrovirals, potentially complicating initial clinical management and the accurate measurement of viral load [154]. Due to variations in virulence and differing susceptibility or resistance patterns to antiviral therapies, the spread of non-B clades should be monitored and considered in future studies, particularly in European countries [155,156]. Viral mutations can progressively occur after the peak of viremia in acute HIV infection [20].

Early treatment limits the virus’s genetic diversity in the blood [27,157] and gut-associated lymphoid tissue (GALT), thereby potentially enhancing the immune response by reducing epitope variation, which otherwise lowers the effectiveness of specific anti-HIV responses [158]. This low genetic diversity, in contrast to chronically treated patients, is maintained under treatment [30]. Treating patients during AHI prevents viral evolution and the accumulation of defective proviruses, as observed in post-treatment controllers (PTCs) [157]. While there are ongoing discussions among scientists regarding the significance of integrated defective proviruses, it cannot be denied that they may contribute to HIV pathogenesis by producing viral RNAs and proteins that drive persistent immune stimulation, leading to lymphocyte activation and inflammation [159,160]. Viral variabilities, both quantitatively and qualitatively, during the course of an HIV infection are important pathogenetic considerations when aiming to reduce the reservoir. The investigation of additional predictive markers, especially through virological and immunological evaluations, remains a pressing challenge during acute HIV infection [161].

## 6. Key Immunological Considerations in Acute HIV Treatment

By inhibiting viral replication, early treatment during AHI effectively reduces immune activation [30,133,162], can abrogate the HIV-induced cytokine storm [163], and may help limit the systemic spread of viral products [164,165]. This normalization may lower the risk of disease progression compared to patients who are treated later [166,167]. However, in monkey models, lymphoid tissue activation and germinal center hyperplasia persist, indicating ongoing viral replication in HIV reservoir cells [168]. Additionally, low-grade inflammation continues in two-thirds of people living with HIV (PLWH) on antiretroviral therapy (ART), indicating a persistent inflammatory signature [169], even when ART is initiated during AHI. This sustained inflammation may predispose PLWH to cardiovascular and other comorbidities, particularly if treated during chronic infection [170]. Chronic immune activation in HIV likely stems from various factors, including HIV’s direct cytopathic effects, the release of pro-inflammatory cytokines, loss of regulatory T cells, viral co-infections (e.g., cytomegalovirus), and microbial translocation from compromised gut barriers [18]. This widespread inflammation may also impact the central nervous system (CNS), where elevated neuroinflammatory markers like neopterin have been observed [171]. While early ART initiation can reduce cerebrospinal fluid inflammation within six months, inflammatory markers in the blood often remain elevated, highlighting the need for further research into early ART’s potential role in limiting HIV-associated effects on the brain [33].

ART during AHI also leads to a steady gain in CD4+ T-cell count and rapid viral suppression (<50 copies/mL) [128,129,172,173]. CD4+ and CD8+ T-cell functions are preserved [174,175,176] with the maintenance of HIV-specific CD4+ T-cell help, even when treatment is initiated at later stages of AHI [72]. However, mucosal CD4+ T cells are better conserved when treatment begins in early AHI [165,177]. This is similar to what is observed in HIV controllers [178], whereas in patients with chronic HIV, ART improves mucosal CD4+ T-cell differentiation but cannot prevent the persistent depletion of total CD4+ T cells [164]. Early treatment can partially restore gut CD4+ T cells, though some degree of functional impairment may persist. Starting ART before Fiebig stage I/II can also prevent both the quantitative and qualitative loss of Th17 cells, which are crucial for maintaining mucosal barriers [89]. Furthermore, the deactivation of CD8+ T cells with favorable functional profiles allows for memory T-cell proliferation and function [179], especially when ART is initiated before peak viremia [180]. These memory T cells, which are absent in untreated or later-treated individuals, are crucial for virologic control and are associated with viral clearance in SIV infection [181]. In macaque-modeled studies, early ART promotes post-treatment controller (PTC) status by expanding central memory CD8+ T cells with enhanced survival and proliferation [182]. CD4/CD8 recovery is faster and more robust in those treated during AHI [183,184], particularly at the very early stages (Fiebig stage I). However, persistent immune dysfunction is reflected in a reduced CD4/CD8 ratio [40] which is associated with ongoing chronic inflammation [185].

Additionally, AHI treatment restores natural killer and dendritic cell numbers [186,187] and preserves B-cell responses to HIV and non-HIV antigens by maintaining functional follicular T helpers and B cells [101,188]. Surprisingly, extremely early ART initiation may prevent the development of an antiviral response altogether, as observed in rare non-reactive cases detected with fourth-generation assays [189]. However, such early treatment does not prevent viral rebound after ART discontinuation [190]. In clinical practice, very early diagnosis is not so feasible, and patients are generally treated around four weeks post-infection [11,128]. Optimal immune priming, balancing early ART benefits, and proper immune response development are crucial for maximizing the advantages of early treatment, as observed in PTC studies [182].

The benefits of early treatment may not always persist after treatment interruption, supporting the rationale for discontinuing treatment only under strictly controlled conditions or within therapeutic trials. Lastly, qualitative and quantitative differences in the reservoir between patients treated during AHI versus chronic HIV infection highlight the multiple advantages of initiating treatment during AHI. The main benefits of initiating treatment during acute HIV infection compared to late treatment are shown in Table 1.

## 7. Clinical Implications and Challenges in Acute HIV Treatment

The immunovirological impact of treatment during symptomatic AHI helps reduce the intensity and duration of symptoms, decreases the frequency of opportunistic infections, and lowers mortality and morbidity in these patients [167,175,191]. Interestingly, women who initiate ART during AHI exhibit a stronger immunovirological response compared to men, which may provide them with additional protection against adverse clinical events and premature aging [192]. In addition, treating AHI has a neuroprotective effect by limiting immunological processes that are deleterious to the central nervous system (CNS). It likely arrests the effects of HIV on the CNS, offering a better neuroprotection when compared to initiation during chronic infection [33]. Furthermore, immune recovery with reduced inflammation [185] and relative preservation of immune cells [176] allow a significant improvement in patient quality of life [172]. Nevertheless, inflammation persists even after starting ART and achieving undetectable plasma HIV RNA levels [169]. Factors that may contribute to chronic immune activation include the direct cytopathic effect of HIV, the production of pro-inflammatory cytokines by innate immune cells, the depletion of regulatory T cells, viral co-infections like cytomegalovirus (CMV), and the translocation of microbial products through damaged intestinal mucosal barriers [18]. Moreover, defective HIV proviruses can trigger immune responses through their RNA transcripts and viral proteins, which form virus-like particles. This continuous production of HIV-1 proteins from defective proviruses, even without active replication, contributes to persistent immune activation, despite undetectable HIV-1 levels [193]. Interestingly, studies in PTCs have shown that the combination of low viral diversity and defective provirus levels, along with specific host and immune factors, likely contributes to the control of HIV infection in PTCs who were treated during AHI, highlighting another benefit of treatment at the time of AHI [157]. Also, despite persistent immune activation, long-term clinical benefits, such as reductions in cardiovascular events, cancer, and HIV-related complications, are expected in patients treated during AHI and should be further explored in the coming years [33].

Several studies conducted in European or North American countries suggest that newly infected patients are a major source of transmission, maintaining the epidemic within risk groups [194]. These studies have detected transmission of new viral clusters from patients with acute or early infection [25,195]. The question of treatment as prevention (TASP) is particularly significant in AHI with high levels of genital and plasma viral load [196]. The transmission risk during AHI is 26 times greater than that of the chronic stage [2] and estimated to be responsible for up to 50% of all new infections [195]. The risk of sexual HIV-1 transmission is also closely correlated with the level of HIV-1 RNA in genital secretions [197], which follows the dynamic of HIV-1 RNA in blood [198,199]. Early treatment during AHI facilitates the purging of viral particles and infected cells in the genital compartment [47,48]. Consequently, drug pharmacokinetics may be a factor to consider for treatment regimens, including drugs that concentrate in the genital tract of men and women. Integrase inhibitors are among the most effective drugs for rapidly decreasing viral load, including within the genital tract [48]. In addition, the ‘chemsex’ practice of using drugs to enhance and extend sexual experiences has become increasingly prevalent in urban communities, particularly among men who have sex with men (MSM) [200]. This behavior significantly increases the risk of HIV transmission especially for patients with AHI [200,201]. Moreover, chemsex is associated with increased frequency of sexual partner changes and decreased condom use, due to drug-induced loss of inhibition and reduced engagement in HIV testing [202]. A higher prevalence of chemsex of 29% has been reported in HIV+ MSM [203,204]. Additionally, individuals involved in chemsex tend to have less consistent adherence to HIV treatment, further exacerbating the risk of untreated acute infection or therapeutic non-adherence and complicating efforts to prevent HIV transmission [202,203]. Patients who engage in chemsex should be incorporated into preventive and educational programs aimed at reducing the occurrence of high-risk sexual behaviors and STIs [203].

More recently, pre-exposure prophylaxis (PrEP) with emtricitabine/tenofovir is a new high-efficacy tool to reduce HIV incidence among MSM when adherence is optimal [205,206,207]. Despite its high efficacy, non-adherence to daily PrEP remains a significant issue. A study of 210 gay men engaged in PrEP in California and New York revealed that over two-thirds missed doses, with an average of four to five missed doses in the past month. Socio-behavioral factors, such as attitudes, perceived control, and social barriers, contributed to non-adherence [208]. Enhancing adherence could help reduce new HIV infections. Healthcare providers should play a major role in promoting adherence through strategies that encourage habit formation, ultimately helping to reduce transmission risks particularly in high-risk groups [208]. In addition, there is a context of declining condom use in the general MSM population, including those living with HIV, evidenced by the concomitant increase in the incidence of bacterial STIs since the early 2000s, notably in France [209,210,211]. Although rare, HIV infections can occur despite high adherence to PrEP. With the anticipated broader use of long-acting PrEP, new challenges are likely to arise. Prompt identification of these infections should be integrated into PrEP follow-up to minimize the risk of overlooked HIV infections and exposure to suboptimal antiretroviral concentrations [211].

To date, some studies have evaluated the immunovirological efficacy of treatments and observed that virological rebounds following treatment interruption increase the risk of transmission, as reported in the Swiss cohort study [212] and the OPTIPRIM ANRS-147 clinical trial, in which a case of viral transmission occurred one month after treatment interruption [128]. Finally, the significantly small proportion of patients treated during AHI who achieve PTC [145] strongly argues against treatment cessation outside of clinical trial settings. In this context, a functional cure strategy aims to reduce the viral reservoir and enhance immune control, allowing HIV suppression without ART. Over the years, several approaches have emerged, including ‘shock-and-kill’, which reactivates latent HIV with latency reversal agents before eliminating infected cells, and ‘block-and-lock’, which prevents viral reactivation. Therapeutic vaccination, the use of PD-1 inhibitors and Toll-Like Receptor agonists to enhance HIV-specific CD8+ T-cell responses to target and eliminate infected cells, and adoptive chimeric antigen receptor (CAR) T-cell therapy are also being investigated as potential strategies [213]. Another strategy for eliminating the virus involves the use of HIV-1-specific broadly neutralizing antibodies (bNAbs), which target a conserved region of the HIV envelope, allowing them to neutralize a broad range of HIV strains, and they are being actively studied to control HIV replication in patients treated during AHI [214,215,216]. The simplification of treatment during the chronic phase, aimed at reducing long-term side effects while preserving immunovirological efficacy, remains a subject of ongoing debate. Nevertheless, another challenge lies in defining the optimal timing for simplification, such as the use of dual therapy, particularly for patients who initiated and maintained early treatment after AHI.

## 8. Conclusions

Despite decades of HIV prevention efforts, the HIV epidemic persists, underscoring the critical challenge of detecting infections at the earliest stage. Improving awareness, strengthening screening programs, and ensuring quick access to care—particularly for high-risk populations—are essential. Initiating antiretroviral therapy during acute HIV infection not only helps limit transmission within at-risk populations but also provides substantial immunological and virological benefits at the individual level.

Innovative strategies, including clinical studies exploring ART interruption and immune-based interventions such as broadly neutralizing antibodies (bNAbs), present promising avenues toward a functional cure. Prioritizing patients treated during the acute phase of HIV infection for these studies could further progress toward the development of more effective treatments. Taking these factors into account will accelerate progress in combating the HIV epidemic and advancing toward the goal of a functional cure.

## Figures and Tables

**Table 1:** Main benefits of initiating treatment during Acute HIV Infection compared to late treatment.

**Patient treated at the time of acute infection**	**Patient treated at the chronic phase**
Transmission is typically homogeneous, involving a single virion with dominant CCR5 viral tropism	Both R5 and X4 variants emerging during infection and associated with rapid disease progression
Preventing escape mutations and limiting viral diversity Less defective proviral gene insertions, limiting immune activation	Large viral diversity
Lower virologic set point	
Best reduction of residual viremia	
**The treatment quickly restores and protects the immune system:** Protection of the different polyfunctionalities of TCD4+ and TCD8+, limit apoptosis by reducing activation and viral diversity	**More limited immune restoration:** From the surviving TCD4+ and TCD8+ clones to achieve the different polyfunctionalities
**Major difference in the quantity and quality of the reservoir**	
**Short Half-Life Reservoir:** TTM infection and TCM protection Better reduction in total HIV-1 DNA and integrated HIV-DNA Prevents the establishment of the stable form of integrated proviral HIV-1 DNA that is less prone to elimination	**Long Half-Life Reservoir:** TCM Infection Blood reservoirs are mainly composed of stable proviruses in long-lived quiescent cells and are unaffected by ART
**Control of replication in post-treatment, VISCONTI:** A preserved immune system that allows effective control of replication and suppression of the viral reservoir after treatment discontinuation.	**No control of replication:** An immune system unable to control replication after treatment discontinuation.
**Optimal conditions for future cure trials**	
